# A Promising Candidate for a Lassa Fever Vaccine

**DOI:** 10.1371/journal.pmed.0020196

**Published:** 2005-06-28

Lassa fever, a viral hemorrhagic fever caused by the Lassa virus and commonly transmitted by its rodent host, is endemic in certain areas of West Africa, where several hundred thousand people are estimated to be infected each year. The disease is asymptomatic or mild in approximately 80% of infected patients, but the remaining 20% have severe multisystem disease. Estimated overall mortality is 1%–2%.

Death rates are particularly high for women in the third trimester of pregnancy, and for fetuses, about 95% of which die in the uterus of infected pregnant mothers. The most common complication of Lassa fever is deafness. Various degrees of deafness occur in approximately one-third of cases, and in many cases hearing loss is permanent. Disease severity does not seem to affect this complication: deafness may develop in mild as well as in severe cases.

Lassa fever remains a serious challenge to public health in West Africa, threatening both local residents in rural areas and those who serve them, particularly medical care providers. Ribavirin, an antiviral drug, has been used successfully in Lassa fever patients, but it needs to be given early and is not readily available in the infected areas. Given the ecology of the rodent host and conditions in the endemic area, a vaccine is mandatory for control. Lassa vaccine initiatives have suffered from a lack of funding in the past, but bioterrorism and recent importation of the disease to the United States and Europe have brought new resources to Lassa virus science.

Early attempts to develop a Lassa fever vaccine in the 1980s focused on killed pathogens, which caused a strong humoral response but failed to protect nonhuman primate test animals. Subsequently, recombinant vaccines used vaccinia vectors carrying different combinations of structural Lassa proteins. Some of these protected 90% of nonhuman primates from a lethal challenge in the absence of a strong humoral response, suggesting that cellular responses are important for protection.

Use of vaccinia vectors in humans is problematic, especially in areas where HIV infection is common—immune-suppressed individuals can develop serious skin lesions—and several alternative vaccines based on other vectors as well as harmless vaccinia ones are under development. Thomas Geisbert and colleagues now report promising results with a replication-competent vaccine based on attenuated recombinant vesicular stomatitis virus vectors expressing the Lassa viral glycoprotein. A single intramuscular vaccination protected all four vaccinated cynomolgus macaques against a lethal challenge of a particular Lassa strain, while two control monkeys that had received empty vector died after injection with the same dose of virus.

These are encouraging results, but future larger studies will need to assess the duration of protection and demonstrate the safety of this replication-competent vaccine. Another crucial question is how quickly vaccinated individuals acquire protection, and thus whether the vaccine would be suitable for creating a ring of vaccination around an outbreak zone, the most likely early application of a promising candidate vaccine. In addition, there are at least four different strains of the Lassa virus, and an ideal vaccine should provide protection across all strains. Finally, conducting trials in endemic areas, many of which lack political stability, remains a serious challenge. [Fig pmed-0020196-g001]

**Figure pmed-0020196-g001:**
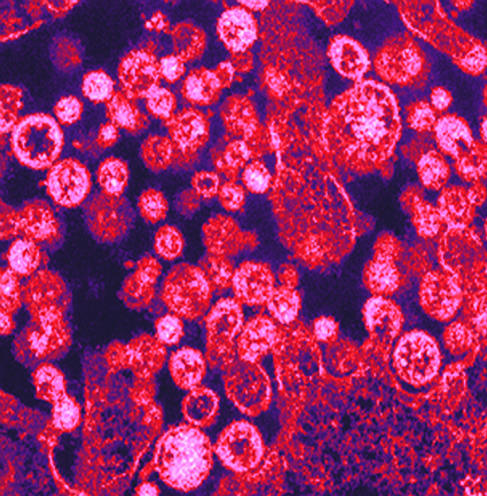
Color-enhanced transmission electron micrograph of Lassa virus particles

